# The effect of temperature and retention time on methane production and microbial community composition in staged anaerobic digesters fed with food waste

**DOI:** 10.1186/s13068-017-0989-4

**Published:** 2017-12-14

**Authors:** John Christian Gaby, Mirzaman Zamanzadeh, Svein Jarle Horn

**Affiliations:** 1 Faculty of Chemistry, Biotechnology and Food Science, Norwegian University of Life Sciences, Ås, Norway; 2 Department of Environmental Health Engineering, School of Public Health, Tehran University of Medical Sciences, P.O. Box 14155-6446, Tehran, Iran; 3 Department of Civil and Environmental Engineering, University of Waterloo, 200 University Avenue West, Waterloo, ON N2L 3G1 Canada

**Keywords:** Temperature-phased anaerobic digestion, Retention time, Biogas, Microbial community analysis, Biofuel, Methane

## Abstract

**Background:**

Food waste is a large bio-resource that may be converted to biogas that can be used for heat and power production, or as transport fuel. We studied the anaerobic digestion of food waste in a staged digestion system consisting of separate acidogenic and methanogenic reactor vessels. Two anaerobic digestion parameters were investigated. First, we tested the effect of 55 vs. 65 °C acidogenic reactor temperature, and second, we examined the effect of reducing the hydraulic retention time (HRT) from 17 to 10 days in the methanogenic reactor. Process parameters including biogas production were monitored, and the microbial community composition was characterized by 16S amplicon sequencing.

**Results:**

Neither organic matter removal nor methane production were significantly different for the 55 and 65 °C systems, despite the higher acetate and butyrate concentrations observed in the 65 °C acidogenic reactor. Ammonium levels in the methanogenic reactors were about 950 mg/L NH_4_^+^ when HRT was 17 days but were reduced to 550 mg/L NH_4_^+^ at 10 days HRT. Methane production increased from ~ 3600 mL/day to ~ 7800 when the HRT was decreased. Each reactor had unique environmental parameters and a correspondingly unique microbial community. In fact, the distinct values in each reactor for just two parameters, pH and ammonium concentration, recapitulate the separation seen in microbial community composition. The thermophilic and mesophilic digesters were particularly distinct from one another. The 55 °C acidogenic reactor was mainly dominated by *Thermoanaerobacterium* and *Ruminococcus*, whereas the 65 °C acidogenic reactor was initially dominated by *Thermoanaerobacterium* but later was overtaken by *Coprothermobacter*. The acidogenic reactors were lower in diversity (34–101 observed OTU_0.97_, 1.3–2.5 Shannon) compared to the methanogenic reactors (472–513 observed OTU_0.97_, 5.1–5.6 Shannon). The microbial communities in the acidogenic reactors were > 90% Firmicutes, and the Euryarchaeota were higher in relative abundance in the methanogenic reactors.

**Conclusions:**

The digestion systems had similar biogas production and COD removal rates, and hence differences in temperature, NH_4_^+^ concentration, and pH in the reactors resulted in distinct but similarly functioning microbial communities over this range of operating parameters. Consequently, one could reduce operational costs by lowering both the hydrolysis temperature from 65 to 55 °C and the HRT from 17 to 10 days.

**Electronic supplementary material:**

The online version of this article (10.1186/s13068-017-0989-4) contains supplementary material, which is available to authorized users.

## Background

The anaerobic digestion process allows the recovery of energy as methane, which may be used for power and heat production, or as transport fuel. In 2013 the European Union produced 13.4 million tons of oil equivalents from biogas [1]. A number of organic substances of varying compositions may be used for biogas production, and in particular, household and commercial food waste is widespread, abundant, and energy rich. There is high-potential biogas yield from food waste because of its high energy content and the presence of easily degradable carbohydrates and proteins [2]. An estimated 1.3 billion tons of food is wasted annually across the globe at all stages of production and supply. In middle- and high-income countries a larger portion of food is wasted at the consumer level, like for example, in Europe and North America where 95–115 kg/year is wasted per capita vs. the 6–11 kg/year wasted in sub-Saharan Africa and South/Southeast Asia [3]. Food waste is a source of reclaimable energy, and the anaerobic digestion of food waste is the most sustainable way to recover that energy while reducing environmental impacts of waste that would otherwise be landfilled or incinerated [4, 5].

During anaerobic digestion (AD), hydrolysis is the first step in which long-chain organic molecules are broken down by hydrolytic bacteria into their constituent monomers. Acidogenic bacteria then ferment the monomers mostly into volatile fatty acids (VFAs) such as acetate, propionate, and butyrate. Short-chain VFAs (i.e., propionate, butyrate, and valerate) are converted into acetate via the process termed acetogenesis. Finally, the acetate, CO_2_, and H_2_ generated in the previous steps are used by methanogens to produce methane gas.

Several anaerobic digestion (AD) configurations have been used so far in order to enhance methane production, including single-stage AD, staged AD, leach-bed reactors, and hybrid anaerobic digesters [6, 7], among which staged or multiple-staged digestion was reported to be a promising technology because of enhanced performance and flexibility in operation [8, 9]. In the staged digestion concept, the purpose is to separate and control the hydrolysis–acidogenesis processes from the methane production step due to the physiological and kinetic differences of these microbial groups [10].

Most research on staged anaerobic digestion has focused on comparing its performance with single-stage anaerobic digestion or enhancing process performance [8, 11]. While the performance advantages of multi-staged anaerobic systems are now recognized, the microbial taxa involved, their function, and the compositional changes they undergo in response to different operating parameters [12, 13] are poorly understood. Thus, further research is needed to link the myriad microbial functions that occur in each stage with their respective taxa, and it is therefore of great interest to study the microbial ecology of two-staged anaerobic digestion of food waste.

Staged anaerobic digestion systems typically consist of a hydrolytic thermophilic reactor followed by a mesophilic methane producing reactor. High temperature in the first-stage reactor is advantageous because it increases hydrolysis rate and allows for a smaller reactor volume. For the second-stage reactor where methane production takes place, mesophilic conditions usually result in more stable biogas production. Some hydrogenotrophic methanogenesis in the hydrolysis reactor is beneficial since this may improve acidogenesis and solubilization due to the removal of H_2_ produced from these reactions. It is indicated in the literature that increasing temperature from 55 to 70 °C resulted in a better performance of hydrogenotrophic methanogens [14, 15].

Another important operational parameter that influences anaerobic digestion process and microbiology is hydraulic retention time (HRT). Typical HRTs used in the 1st reactor of staged digestion systems are 2–4 days. It has also reported that operation of the methanogenic stage at HRTs lower than 8–10 days may lead to instability of methanogenic process [16–18].

The flexibility in operating each stage of staged anaerobic digesters (i.e., changing variables like temperature, organic loading rate, and hydraulic retention time) makes it possible to study the impact of these parameters on the microbial ecology in each stage. We studied the effect of altering two operational parameters in a 2-stage reactor system, which consisted of an initial thermophilic hydrolysis/acetogenesis followed by a mesophilic methanogenesis stage. First, we examined the effect of operation at 55 vs. 65 °C in the first stage. Second, we examined the effect of reducing the total hydraulic retention time (HRT) from 20 to 13 days by reducing the HRT in the second stage from 17 to 10 days. We characterized reactor performance, biogas production, and the microbial community composition as they responded to these parameter changes. Moreover, we identified those process parameters which could account for the differences in microbial community composition including changes in abundance of specific microbial taxa.

## Methods

### Digesters operation

The experimental apparatus consisted of two sets of staged, laboratory-scale digestion systems (Belach Bioteknik, Sweden). Two completely mixed reactors in series were used (Additional file 1: Image S1) to set up the following staged digestion systems: digestion system 1 with a thermophilic 1st stage (TDS1) and a mesophilic 2nd stage (MDS1), and digestion system 2 with a thermophilic 1st stage (TDS2) and a mesophilic 2nd stage (MDS2). After a change in total HRT (from 20 to 13 days, see below), these systems were called digestion system 3 (TDS3 and MDS3), and digestion system 4 (TDS4 and MDS4). Herein we sometimes refer to both coupled stages as a single Digestion System (e.g., DS1 is TDS1–MDS1). Two small reactors with 2.5 L working volume were used as first stage (one at 55 °C, one at 65 °C) whose effluent was transferred to the 2nd reactors. The second-stage digesters consisted of larger reactors with 6 L working volume and were operated at 37 °C. The anaerobic digestion systems were run in two experimental periods resulting in a total of 4 DS (Additional file 2: Figure S1). In the first experimental period, the digesters (DS1 and DS2) were operated for 150 days (from March 3 to July 31, 2015) while reaching steady state at an overall system HRT of 20 days, for which the first and second stages were operated at 3 and 17 days HRT, respectively. During the last 30 days of both experimental periods, the reactors displayed steady-state conditions based on biogas production, and following steady-state confirmation, samples were taken for complete chemical and microbiological analysis for 3 consecutive weeks. Thereafter, the HRT in the second-stage reactors (DS3 and DS4) was reduced from 17 to 10 days by daily feeding of 0.6 L of the first-stage effluent, and withdrawing the same amount out of the second stage. Hence, the overall HRT of the digestion system was reduced to 13 days. The second stage of the experiments was performed for about 140 days (from August 1 to December 21, 2015). Throughout the whole experimental period, the two systems were identical except for the first-stage temperature which was 55 °C in TDS1 and TDS3 but 65 °C in TDS2 and TDS4. The temperature and mixing of the systems were controlled using BioPhantom software (Belach Bioteknik, Sweden). The inoculum to seed the digesters was taken from lab-scale digesters that were run at 37 and 62 °C for more than a year and exclusively fed with the same pasteurized food waste as used in this study. The characteristics of the food waste are given in Table 1.

**Table 1:** Characteristics of food waste used to feed the phased digestion systems

Parameter	Unit	Value
Total solids	%	30.06 ± 0.68
Volatile solids	%	24.87 ± 0.54
VS/TS		0.93
TCOD	g/L	50.47 ± 13.84
TCOD/VS		2.00
SCOD	g/L	15.60 ± 0.60
TAN	mg/L	55.50 ± 8.90
pH		7.02 ± 0.22

Original food waste was diluted using tap water to achieve the target VS concentration for feeding the first-stage digesters

*TAN* total ammonia nitrogen

### Chemical analysis

Various chemical parameters were measured in the digestion systems (Additional file 3: Spreadsheet 1). An aliquot of the samples was centrifuged at 10 K rpm and then filtered before analysis of pH, NH_4_^+^, and alkalinity. The pH was determined using an Orion pH meter (Thermo Scientific, USA). NH_4_^+^ was measured using an ammonium cell test and following the manufacturer’s instructions (Merck, USA). Chemical Oxygen Demand (COD) and volatile solids (VS) were determined according to Standard Methods (APHA, 1998). Alkalinity was measured by titration according to the Nordmann Method [19].

VFAs were quantified on a Dionex Ultimate 3000 HPLC with ZORBAX Eclipse Plus C18 column. The HPLC was equipped with a UV detector measuring at 210 nm wavelength. Samples for VFA analysis were centrifuged at 15,000 rcf for 600 s to pellet solids, then 95% H_2_SO_4_ was added in a 1:100 ratio (v/v) to an aliquot of the supernatant and mixed. The solution was again centrifuged for 10 min at 15,000 rcf, and a 200 µL aliquot portioned out for HPLC analysis. Quantification was made by reference to standard curves of five dilutions of acetic, propionic, butyric, valeric, and iso-valeric acids. Chemical data were explored and visualized with the Orange3 data mining software [20] and in the R statistical analysis software [21] using the ‘stargazer’ [22] and ‘tables’ [23] packages. Principal component analysis of process parameters was performed in the software PAST [24] using the correlation matrix, which normalizes the data.

### Microbial community analysis

Samples were taken from the digester effluents at multiple time points (Additional file 3: Spreadsheet S1). The samples were stored frozen at − 20 °C until further processing. DNA was extracted from 1.0 mL of reactor fluid using a CTAB-based, indirect extraction protocol (Additional file 4: Protocol S1) adapted from a previously described method [25, 26]. 2.5 µL of extracted DNA was used as template in a PCR reaction to amplify the 16S rRNA gene using the primers Pro341F (5′-CCTACGGGNBGCASCAG-3′) and Pro805R (5′-GACTACNVGGGTATCTAATCC-3′) [27]. The 25 µL PCR reactions consisted of 1X iProof High-Fidelity Master Mix (Bio-Rad Laboratories, Hercules, CA, USA) and 0.2 µM of each of the primers Pro341F and Pro805R. The PCR thermal cycling consisted of a hot start step at 98 °C for 180 s followed by 25 cycles of 98 °C for 30 s, 55 °C for 30 s, 72 °C for 30 s, and then a final 72 °C extension step for 300 s. PCR products were then barcoded using the Nextera XT indexing kit (Illumina, San Diego, CA, USA) according to the manufacturer’s protocol. Barcoded samples were purified using Agencourt AMPure XP magnetic beads (Beckman Coulter, Brea, CA, USA) and quantified using the Qubit dsDNA BR Assay Kit (Thermo Fisher Scientific, Waltham, MA, USA) prior to equilibration of each barcoded sample to an equimolar concentration. Samples were sequenced on the Illumina MiSeq using the Reagent Kit v3 for 2 × 300 paired-end sequencing (Illumina, San Diego, CA, USA).

Sequence processing and data analysis were performed on an ASUS laptop with 2.6 GHz quad-core Intel Core i7-6700HQ CPU and 16 GB RAM running Bio-Linux 8 [28]. First, the paired-end reads were merged using PEAR version 0.9.6 [29] specifying a maximum assembly length of 575, a minimum assembly length of 400, and a minimum overlap of 50 nucleotides and disabling the statistical test. The merged sequences were quality filtered using PRINSEQ [30] by removing sequences that had at least one base with a quality score below 20 or that had a mean quality score below 30. Next, primers were trimmed using the trim.seqs function of MOTHUR [31] version 1.36.1. Chimeric sequences were identified using the de novo chimera checking implemented in the USEARCH61 algorithm [32, 33] as implemented in the identify_chimeric_seqs.py script of QIIME [34] version 1.9.1. The chimeric sequences were removed using the QIIME script filter_fasta.py. Then, the FASTA headers for each sequence were reformatted as the sample name followed by the within-sample sequence number (e.g., 35_589 for sample 35, sequence 589), and all sequences were merged into a single file prior to further processing with QIIME. The QIIME workflow script pick_open_reference_otus.py was used to cluster sequences with the USEARCH61 algorithm [32, 35] at the standard 97% sequence similarity that approximates a species cutoff. Then, the QIIME workflow script core_diversity_analyses.py was used to output alpha and beta diversity analyses as well as stacked bar plots of taxonomic diversity. Rarefaction to 18,000 sequences was specified in the diversity analysis. We used the software STAMP [36] to identify taxa with significantly different abundances between reactor comparisons. We further analyzed the dataset by oligotyping [37] and Minimum Entropy Decomposition [38]. A bipartite oligotype network was visualized in Gephi [39] using the Fruchterman–Reingold algorithm with samples colored according to experiment and reactor and edge thickness weighted by number of sequences of an oligotype found in a sample.

### Sequence data storage and access

Sequence data were deposited in the NCBI Sequence Read Archive under BioProject Accession PRJNA407631 with corresponding BioSample Accessions SAMN07691008 to SAMN07691054 and SRA Accessions SRR6067579 to SRR6067625.

## Results

### Performance of staged anaerobic digestion systems with a HRT of 20 days: TDS1–MDS1 and TDS2–MDS2

Our first experimental comparison was focused on the effect of a 10 °C difference in temperature during the thermophilic hydrolysis/acidogenesis stage (TDS1 at 55 °C and TDS2 at 65 °C). However, the greatest distinction was of course between the digestion stages (i.e., TDS1 vs. MDS1 and TDS2 vs. MDS2) which differed in temperature by 18 °C for TDS1–MDS1 and 28 °C for TDS2–MDS2. The thermophilic reactors also had different HRTs and organic loading rates (OLRs) as compared with the mesophilic reactors (Table 2). Hence, the reactor environmental conditions varied with the greatest differences between the stages (Table 2; Additional file 8: Figure S5). TDS1–MDS1 generated about 3% more methane (Table 2) than the digestion system 2 (TDS2–MDS2). However, both digestion systems showed comparable performance in terms of VS and COD removal and methane production (Table 2). Comparing the separate stages of the digestion systems, it is clear that VFA concentrations were much higher in the first-stage reactors, and interestingly butyrate (6161 ± 339 and 6947 ± 641 mg/L) was higher than acetate (2607 ± 178 and 3744 ± 390 mg/mL) in these reactors (Table 2). The accumulation of VFAs in the first-stage reactors resulted in low pH values, being 5.70 and 5.55 for TDS1 and TDS2, respectively. This is well below the optimal pH range for activity of acetoclastic methanogens. The low amount of methane generated in TDS1 (93 ± 22 mL/day) and TDS2 (39 ± 17 mL/day) was likely produced by the hydrogenotrophic methanogen *Methanothermobacter* (Fig. 5) as well as syntrophic acetate oxidizers that can resist a wider range of pH [13, 40]. The accumulation of VFAs and low methane production agreed with high concentrations of soluble COD in the first-stage reactors (Table 2). Acetate concentrations were higher in the 65 °C reactor (TDS2; 3744 ± 390 mg/L) than the 55 °C reactor (TDS1; 2607 ± 177 mg/L). This was also the case for butyrate (6947 ± 641 vs. 6161 ± 339 mg/L), while propionate was higher in TDS1 than TDS2 (143 ± 23 vs. 32 ± 7.6 mg/L). Valerate was comparable for the two temperatures (280 ± 198 and 296 ± 57 mg/L).

**Table 2:** Operating parameters and performance variables (mean ± standard deviation) for the 8 anaerobic digesters during steady state

Parameter	Unit	Digesters							
		DS1		DS2		DS3		DS4	
		TDS1	MDS1	TDS2	MDS2	TDS3	MDS3	TDS4	MDS4
HRT	Days	3	17	3	17	3	10	3	10
Temperature	°C	55	37	65	37	55	37	65	37
Methane flow (3-day mean)	mL/day	49 ± 5	3991 ± 307	32 ± 6	3665 ± 158	135 ± 49	7289 ± 1001	446 ± 134	6890 ± 921
Methane flow	mL/day	93 ± 22	3889 ± 583	39 ± 17	3523 ± 284	191 ± 96	8019 ± 517	478 ± 150	7761 ± 579
CH_4_	%	4 ± 4	70 ± 2	2 ± 1	69 ± 2	9 ± 3	70 ± 1	28 ± 5	71 ± 1
pH		5.69 ± 0.06	8.03 ± 0.06	5.55 ± 0.06	7.98 ± 0.09	5.74 ± 0.08	8.09 ± 0.11	6.22 ± 0.08	8.07 ± 0.09
Total ammonia nitrogen	mg/L	121 ± 18	966 ± 84	101 ± 12	915 ± 88	170 ± 16	534 ± 27	256 ± 19	579 ± 25
Free NH_3_	mg/L	0.2	90.2	0.2	76.3	0.3	55.8	2.2	59.0
TCOD	g/L	57.9 ± 30	15.7 ± 12.8	54.1 ± 33.4	16.3 ± 13.5	39.1 ± 1.4	10.5 ± 1.6	44.1 ± 3.9	13.4 ± 1.1
PCOD	g/L	41.6 ± 30.1	14.2 ± 12.9	37.3 ± 33.5	14.8 ± 13.5	24.9 ± 3.1	8.8 ± 1.5	29.1 ± 5.3	11.7 ± 1
SCOD	g/L	16.4 ± 0.6	1.5 ± 0.3	16.8 ± 1.5	1.5 ± 0.3	14.2 ± 2.8	1.7 ± 0.3	15 ± 2.7	1.7 ± 0.2
COD Conc.	g/L	44.3 ± 16.2	10.5 ± 1.3	41.8 ± 16.7	10.7 ± 0.7	39.4 ± 1.1	10.9 ± 1.2	43.0 ± 1.9	13.1 ± 0.7
COD removal	%	78.4 ± 3.8		77.6 ± 4.7		77.0 ± 3.3		71.0 ± 2.0	
Alkalinity	g/L as CaCO_3_	1.5 ± 0.4	6.1 ± 0.7	1.3 ± 0.2	6.2 ± 0.2	1.2 ± 0.4	4.7 ± 0.2	1.7 ± 0.2	5 ± 0.2
FOS/TAC		3.25 ± 1.25	0.06 ± 0.07	3.8 ± 0.46	0.03 ± 0.05	4.65 ± 3.08	ND	2.08 ± 0.28	ND
Acetate	mg/L	2607.4 ± 177.9	19 ± 2.4	3744.1 ± 390	3.8 ± 1.7	2336.5 ± 95.2	ND	2724.7 ± 678.9	ND
Propionate	mg/L	143.4 ± 23.4	1.2 ± 3	32.3 ± 7.6	0.4 ± 0.9	244.4 ± 22.2	ND	237.7 ± 134.3	ND
Butyrate	mg/L	6161.2 ± 339.3	ND	6946.7 ± 641.1	ND	3351.1 ± 178.6	ND	1878.3 ± 1093.9	ND
Valerate	mg/L	296.3 ± 56.9	8.9 ± 8.5	279.5 ± 197.6	13.1 ± 6.1	ND	ND	ND	ND
Volatile solids (VS)	g/L	16.7 ± 1.5	6.3 ± 0.5	17.5 ± 1.6	6.8 ± 0.7	21.6 ± 4.2	7.2 ± 0.7	21.1 ± 2.2	8.3 ± 0.4
VS removal	%	75.2 ± 1.7		74.1 ± 1.0		73.9 ± 2.4		70.1 ± 1.1	

The second-stage reactors MDS1 and MDS2 were functionally comparable. The pH in both second-stage digesters was about 8.0, and they had considerably higher levels of alkalinity and ammonium than the first-stage reactors (Table 2). Accordingly, the individual VFA concentrations (i.e., acetate, propionate, and butyrate) were remarkably decreased compared to the stage-one reactors (Table 2). The majority of the organic matter of food waste (measured as COD) was removed and converted to methane in the second-stage reactors. The overall COD removal was similar (78.4 ± 3.8 and 77.6 ± 4.7%) and methane production was 3889 ± 583 mL/day in MDS1 and 3523 ± 284 mL/day in MDS2 (Table 2).

### Performance of staged anaerobic digestion systems with an HRT of 13 days: TDS3-MDS3 and TDS4-MDS4

Compared to the experiments carried out at a HRT of 20 days, lowering the overall HRT to 13 days did not lead to large changes in performance (Table 2). The methane production in MDS3 (8019 ± 517 mL/day) was slightly higher than in MDS4 (7761 ± 579 mL/day).

In contrast to our first experimental comparison described above, the first-stage TDS4 showed enhanced performance as compared to that of the TDS3. Alkalinity, pH, and ammonia concentration were higher in TDS4 than TDS3 (Table 2). The methane content of the biogas and methane production were also higher in TDS4 than TDS3. The methane production was 478 ± 150 ml/day and the percentage of methane in the biogas was 28% for TDS4, while the corresponding values for TDS3 were 191 ± 96 mL/day and 9%. It should be noted that methane production was clearly higher in both TDS3 and TDS4 than in TDS1 and TDS2, indicating a gradual buildup of methane producing capacity in these reactors over their operation during the two experimental periods.

Soluble COD and VFA concentrations in both TDS3 and TDS4 were high (Table 2), demonstrating efficient hydrolysis and solubilization of particulate organic matter in the digesters. SCOD was 14.2 ± 2.8 in TDS3 and 15 ± 2.7 g/L in TDS4. Acetate concentrations were slightly higher in the 65 °C reactor (2725 ± 679 mg/L) than the 55 °C (2337 ± 95 mg/L). Propionate concentrations were similar in both reactors at around 240 mg/L (Table 2). However, butyrate was higher at 55 °C (3351 ± 179 mg/L) than at 65 °C (1878 ± 1094 mg/L).

The overall COD removal was 77% in DS3 and 71% in DS4. However, the overall methane production was similar for the two systems (191 ± 96 + 8019 ± 517 mL/day for DS3 vs. 478 ± 150 + 7761 ± 579 mL/day for DS4). The pH was effectively the same in the second-stage of the DS3 and DS4 systems (Table 2). However, NH_4_^+^ and consequently alkalinity were slightly higher in MDS4 than MDS3 (Table 2). VFA concentrations were below the detection limit in both reactors MDS3 and MDS4.

### Comparing the overall performance of digestion systems under short and long HRT

**Fig. 1:**
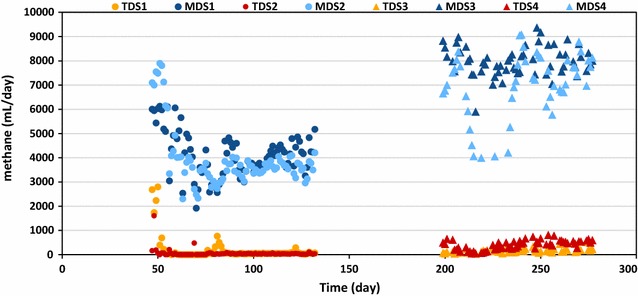
Daily methane production in the 8 digesters during the steady-state periods

**Fig. 2:**
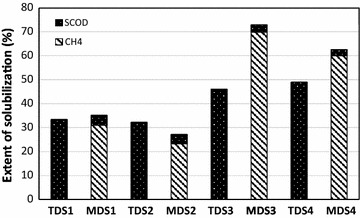
Extent of solubilization based on conversion of particulate COD into soluble products (i.e., soluble COD and CH_4_) in the 8 digesters

The HRT was decreased in the second-stage reactors from 17 to 10 days, and this resulted in a decrease in ammonium concentration from 966 ± 84 (MDS1) and 915 ± 88 (MDS2) to 534 ± 27 (MDS3) and 579 ± 25 (MDS4) mg/L, as well as in the alkalinity from 6.1 ± 0.7 (MDS1) and 6.2 ± 0.2 (MDS2) to 4.7 ± 0.2 (MDS3) and 5 ± 0.2 g/L CaCO_3_ (MDS4). The pH was stable around 8.1 in the four methanogenic reactors (Table 2). VFA concentrations remained low in the methanogenic reactors regardless of retention time (Table 2). When the HRT was lowered, methane production increased (Fig. 1) from 3889 ± 583 to 8019 ± 517 mL/day for MDS1/MDS3 and from 3523 ± 284 to 7761 ± 579 mL/day for MDS2/MDS4. Interestingly, the decreased HRT, which in turn increased the overall OLR of the digestion systems, did not deteriorate the system performance and a similar COD removal was observed in both systems (Table 2). Accordingly, due to the application of higher OLR, methane production increased in both systems as well (Table 2). The solubilization extent, that is the conversion of PCOD into SCOD and ultimately into methane, was calculated for each anaerobic digestion system (Fig. 2). The solubilization extent was almost comparable in the first stages TDS1 (33%) and TDS2 (32%), while the comparison of the values in the second stages demonstrated that MDS1 (35%) showed greater solubilization than MDS2 (27%). A similar observation was seen in the second part of the experiment, that is, the solubilization extent in the first-stage digesters TDS3 and TDS4 was almost similar (46 and 49%, respectively), while the values calculated for the second-stage digesters (73% for MDS3 and 63% for MDS4) were significantly different. Comparison of overall performance of digestion systems in terms of solubilization showed that the increased loading rate under short HRT operation of the digestion systems resulted in greater solubilization extent than those operated at long HRT (Fig. 2).

### Microbial communities

**Fig. 3:**
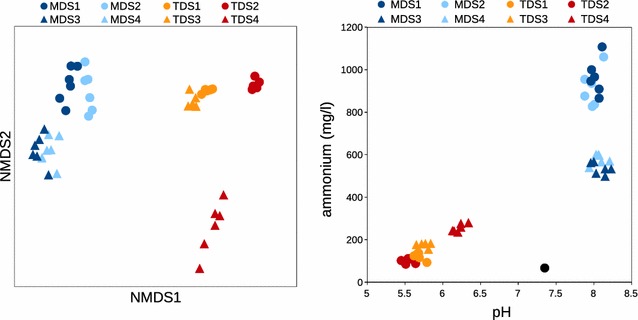
Each reactor has a distinct microbial community composition as shown by 2-dimensional NMDS ordination (left panel), and pH and NH_4_^+^ alone are able to recapitulate a similar pattern of reactor separation (right panel)

**Fig. 4:**
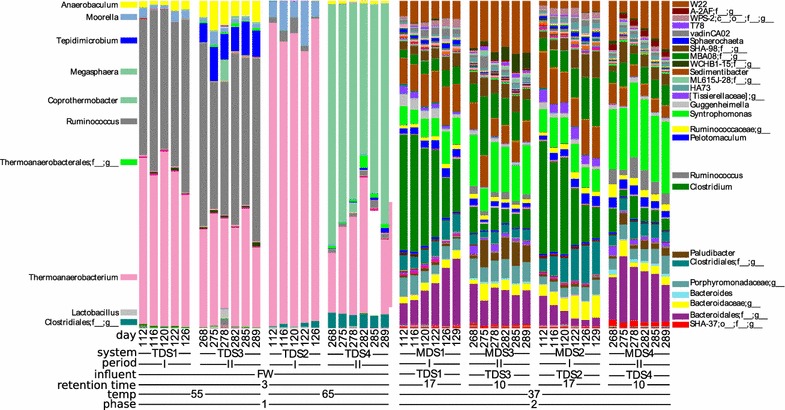
The relative abundance of Bacteria at the genus level for each reactor time point

**Fig. 5:**
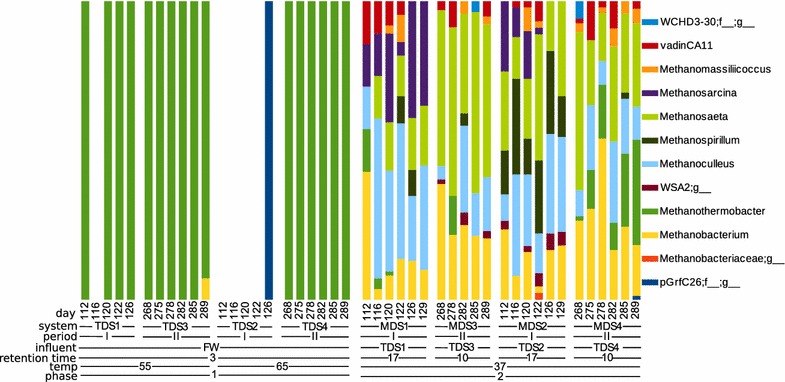
The relative abundance of Archaea at the genus level for each reactor time point

The reactors differed in diversity and community composition with the greatest differences seen between the mesophilic and thermophilic reactors (Figs. 3, 4, 5; Additional file 5: Figure S3, Additional file 6: Figure S4). There was no difference in richness as observed OTU_0.97_ between the 65 °C (49 ± 16) and 55 °C stages (79 ± 26), but the number of OTU_0.97_ in the 37 °C, second-stage reactors (484 ± 36) was 6 to 10 times that of the first stages (Additional file 7: Figure S2). The Shannon diversity index was also higher in the 37 °C reactors (5.41 ± 0.22) than it was for the 65 °C (1.74 ± 0.40) or 55 °C (2.24 ± 0.34) reactors. There was strong separation in the microbial community composition corresponding to temperature differences in the reactors (Fig. 3), and the distinctions in taxa presence and abundance become more apparent at finer levels of taxonomic resolution (Additional file 6: Figure S4; Fig. 4).

An increase in richness was noted in the thermophilic reactors from experimental observation period I to II, whereby the 65 °C TDS2 increased from 34 ± 3 to 63 ± 10 observed OTUs and the 55 °C TDS1 increased from 49 ± 10 to 101 ± 10 observed OTUs (Additional file 7: Figure S2). The thermophilic 65 °C reactor (TDS2 and TDS4) community composition was very distinct between the experimental periods (Figs. 3, 4, 5) due to the emergence and dominance of *Coprothermobacter* during period II (reactor TDS4) which displaced a large portion of the *Thermoanaerobacterium* that dominated in the 65 °C reactor during period I (reactor TDS2; Fig. 4). Firmicutes comprise 95% of the 55 °C and 99% of the 65 °C reactors, and Synergistetes was initially 2.0% of the community in the 55 °C reactor (TDS1) but during the later experimental period (TDS3) tripled to 6.1% (Additional file 6: Figure S4). The 55 °C reactors (TDS1 and TDS3) were dominated by two species, *Ruminococcus* and *Thermoanaerobacterium*, during both experimental periods, but during the second period *Tepidimicrobium* emerged and *Anaerobaculum* increased in abundance (Fig. 4). In the thermophilic reactors, the only uncultivated taxa belonged to 2 orders in the phylum Firmicutes, Clostridiales and Thermoanaerobacterales (Fig. 4).

Mesophilic second-stage reactors exhibited greater evenness than the thermophilic first-stage reactors, and while there were differences in community composition between the different experimental periods, there was a somewhat weaker difference due to influent reactor as either the 55 or 65 °C reactor within an experimental period (Fig. 3). As in the thermophilic reactors, the Firmicutes also dominated in the mesophilic reactor communities with a mean of 60% abundance across all 4 reactors, but other phyla were also abundant across the 4 mesophilic reactors including 20% Bacteroidetes and 8% WWE1 (Additional file 6: Figure S4). The mesophilic reactor communities changed somewhat in composition when their retention time was decreased from 17 to 10 days from experimental period I and II (Figs. 3, 4, 5). The 37 °C reactors had a higher abundance of *Clostridium* during experimental period I, while *Syntrophomonas* and the uncharacterized genus W22 of the phylum WWE1 were more abundant during experimental period II (Fig. 3). There were many uncultivated bacterial lineages present in the mesophilic reactors which are undefined at various taxonomic levels (Fig. 4) including uncultivated taxa affiliated with the WPS-2 phylum, the class SHA-37 (Armatimonadetes), the orders A-2AF (WS6), SHA-98 (Firmicutes), MBA08 (Firmicutes), WCHB1-15 (WS6), ML615J-28 (Tenericutes), Clostridiales (Firmicutes), and Bacteroidales (Bacteroidetes), the families Tissierellaceae (Firmicutes), Ruminococcaceae (Firmicutes), Porphyromonadaceae (Bacteroidetes), and Bacteroidaceae (Bacteroidetes), and the genera HA73 (Synergistetes), T78 (Chloroflexi), vadinCA02 (Synergistetes), and W22 (WWE1).

Methanogenic Archaea were rare (< 1%) in the overall microbial community and were less diverse in thermophilic relative to mesophilic digesters (Fig. 5). The TDS archaeal community consisted of almost entirely *Methanothermobacter*, a hydrogenotrophic methanogen, whereas the MDS community contained more diverse methanogens including the additional hydrogenotrophs *Methanobacterium*, *Methanoculleus*, and *Methanospirillum*, the acetoclastic *Methanosaeta*, and *Methanosarcina* which can undergo either acetoclastic or hydrogenotrophic methanogenesis (Fig. 5). Some methanogens were present transiently or only under certain conditions. Namely, *Methanosarcina* was abundant in MDS mostly during the experimental period I, while *Methanospirillum* was detected mainly during experimental period I in the MDS receiving effluent from the 65 °C TDS although it was also transiently present in MDS1 and MDS3 (Fig. 5).

### Correlation of reactor conditions with the microbial community composition

The microbial community composition can be explained by the set of unique parameter values which defined each reactor environment (Table 2). Indeed, PCA analysis shows the degree of separation of each reactor according to the distinct parameter values (Additional file 8: Figure S5a), with the greatest separation between TDS and MDS systems. The first principal component explains almost 90% of the variation and separates the thermophilic and mesophilic stages, whereas the second principal component explains 6.6% of the variation and separates the mesophilic digesters according to their different performances as a result of the change in HRT during experimental period II (Additional file 8: Figure S5b). In fact, just two variables alone, pH and NH_4_^+^ concentration (Fig. 3b), recapitulate the differences seen in the bacterial community composition for each reactor (Fig. 3a).

## Discussion

The same four reactors were used for experimental periods I and II. Experimental period I examined the effect of 55 vs. 65 °C temperature in the first-stage digesters, and period II examined the effect of lowered HRT in the second-stage digesters. Reactor names were changed to distinguish observations in the new experimental period (e.g., MDS1 became MDS3 during experimental period II).

The performance of the temperature-staged digestion systems for both experimental comparisons was similar (Table 2, Additional file 9: Figure S6). The 10 °C difference in first-stage reactor temperature did not yield significant differences in methane production given that the mean ± standard deviation for the reactor receiving 55 °C effluent was 3889 ± 583 vs. 3523 ± 284 mL CH_4_/day for the reactor receiving 65 °C effluent. The TCOD removal was approximately equal in both DS1 (78.4%) and DS2 (77.6%). When the HRT was lowered in the second-stage reactors from 17 to 10 days, the daily methane production nearly doubled to 8019 ± 517 and 7761 ± 579 mL CH_4_/day for reactors MDS3 and MDS4, respectively. Interestingly, the process was stable at the lower HRT, and thus a lower HRT would require a smaller reactor volume to process an equivalent amount of organic waste to CH_4_ and is consequently more economically favorable. Also, a 55 °C first-stage reactor would require less energy to operate and is thus favorable as compared to a 65 °C reactor. However, the situation might be different if non-heat-treated food waste is used as feedstock.

During both long and short HRTs experiments, the extent of solubilization in the first-stage digesters showed that conversion of particulate organic material (PCOD) into soluble organics (SCOD) was carried out efficiently; however, due to the low retention time, the soluble products were accumulated in the digesters (TDS1 and TDS2) and not converted into methane (Fig. 2). This is attributable to the restricted activity of methanogens, and consequently low methane production, and the dominance of hydrolytic and acidogenic bacteria in the first stages. Interestingly, it was observed that 65 °C in TDS2 did not result in significantly higher solubilization in comparison to that of TDS1 at 55 °C. Operation of the digesters at lower HRT (i.e., 13 days) significantly improved solubilization (Fig. 2). Given that the operating conditions of the first stages did not change during the low HRT experiment, the increased extent of solubilization could be attributed to increased activity of hydrolytic bacteria within the digesters. The solubilization improved significantly in the second-stage digesters as well. The solubilization extents in MDS3 and MDS4 were 2.5 and 2.8 times higher than the corresponding digesters from the high HRT experiments. This improvement in the extent of solubilization was due to the application of a higher organic load in the second-stage digesters. As reported previously [41], the process of hydrolysis during anaerobic digestion can mostly be explained by using a first-order equation with regards to particulate organic matter. Thus, increased organic loading resulted in an increased hydrolysis rate in the second-stage digesters (MSD3 and MSD4). Interestingly, as in the previous sets of experiments having high HRT, it was observed that the overall extent of solubilization in the 55–37 °C digestion system was higher than that of the 65–37 °C one. Since the hydrolysis reaction rate improves at higher temperatures, this discrepancy might be due to the production of some inhibitory materials that resulted in less methane production in TDS4 digester.

There are notable differences between the two experimental periods for variables describing the stage I reactor environments despite having the same feedstock and operational temperature, HRT, and organic loading rate (Table 2, Additional file 9: Figure S6). For example, the 65 °C reactor pH shifted from 5.5 ± 0.06 to 6.2 ± 0.08, and NH_4_^+^ and alkalinity also increased, while total VFA as acetate decreased. This is probably related to the change in microbial community observed between period I and II. The abundance of *Methanothermobacter* increased in the TDS during experimental period II (Additional file 10: Figure S7), supporting that the increased CH_4_ production was due to stabilization of the hydrogenotrophic methanogen population over prolonged reactor operation. Interestingly, during experimental period II, *Coprothermobacter* emerged as the dominant taxa in TDS4 and *Ruminococcus* increased in relative abundance in TDS3 (Fig. 4). In fact, hydrogen may be produced by *Coprothermobacter* [42–44], *Ruminococcus* [45, 46], and *Thermoanaerobacterium* [47, 48], and hence any of these organisms may act to syntrophically contribute hydrogen to *Methanothermobacter*.

The different operational parameters produced a unique environment in each reactor (Fig. 3b; Additional file 8: Figure S5), which resulted in correspondingly distinct microbial communities (Figs. 3, 4, 5; Additional file 5: Figure S3). The operational parameters temperature, HRT, and OLR differed between the separate stages and experimental observation periods. Because there were multiple variables that differed between each reactor, this would confound attempts to attribute the differences in reactor performance and microbial community composition between the stages to any single variable (e.g., pH, NH_4_^+^). In fact, just two parameters, pH and NH_4_^+^ concentration, recapitulate the separation of reactors seen in the microbial community composition (Fig. 3b). Together these variables act as general controls on the microbial community composition. The effect of each of these variables in our reactors is thus described in the following paragraphs.

There is an 18 and 28 °C temperature difference between the mesophilic second-stage reactors and the thermophilic first-stage reactors. At increased temperatures, chemical reactions and biological activity occur at higher rates. In general, thermophilic temperatures result in lower microbial community diversity [49, 50] as was also observed in this study (Additional file 7: Figure S2). For the methanoarchaeal community, which normally exhibits low levels of functional redundancy, this can result in loss of function or instability [51], and indeed it appeared to take the *Methanothermobacter* until the second experimental period to stabilize in the thermophilic reactors (Fig. 5; Additional file 9: Figure S7). There was a higher abundance of Firmicutes in the thermophilic reactors (Additional file 6: Figure S4), and a similar shift to Firmicutes was observed in a study of mesophilic vs. thermophilic reactors degrading household food waste [51]. In a study which increased the temperature of a thermophilic acidogenic pre-treatment reactor from 50 to 60 and then to 65 °C over time [52], a shift was observed from Thermotogae and *Lutispora* (Firmicutes) to *Coprothermobacter* (Firmicutes), which was dominant in our 65 °C reactor during experimental period II (Fig. 4) during which time the ammonium concentration had increased over the levels seen in the experimental period I (Table 2). In a separate study, *Coprothermobacter* comprise 76% of the microbial community in a full-scale, thermophilic, 60 °C food waste reactor with high ammonia levels [53].

The pH is a strong driver of both microbial community composition [54] and function [55], and likewise the influence of pH has been widely observed in other environments like soils [56] and is known from pure-culture studies to be a primary control on microbial growth rate. Indeed, for acetoclastic methanogens as well, there is a narrow pH range for their optimal growth and function with strong inhibition reported below pH 6.2 [57]. In our work the mean pH ranged from 5.5 to 6.2 in the first-stage reactors while the mesophilic reactors were slightly alkaline at pH 8.0 due to the decomposition of N-bearing materials and consequent release of NH_4_^+^ as well as the conversion of VFAs to methane (Table 2).

Substrate composition also affects the composition of the microbial community, and in our work, the reactor feedstock was household food waste. Food waste can be depleted of necessary trace elements required for proper functioning of the microbial community, and this can lead to instability in reactor functioning [58–60]. As a remedy, co-digestion with manure has been shown to stabilize functioning through contribution of trace elements and by acting to buffer pH [61, 62].

The concentrations of NH_4_^+^ and NH_3_ constitute total ammonium nitrogen and are both known to inhibit acetogens and methanogens and thus affect the functioning of biogas reactors [63]. The concentration of free ammonia is known to be the most potent inhibitor and thus its concentration is considered a better predictor of inhibition [63]. Thus, at pH 8.0 and 37 °C in our methanogenic reactors, the free ammonium is expected to be 8–9% of the total nitrogen. The total ammonia nitrogen concentrations that we observed in the MDS1 and MDS2 were 966 and 915 mg/L during observation period I in the second-stage reactors. These ammonium concentrations correspond to a free ammonia concentration of about 90 and 76 mg/L which may slightly inhibit acetoclastic methanogenesis [63, 64].

Methanogens were higher in diversity and relative abundance in the second-stage reactors (Fig. 4). Both acetoclastic and hydrogenotrophic methanogens were present. The acetoclastic methanogens were represented by *Methanosaeta*, and the hydrogenotrophic methanogen by *Methanobacterium, Methanoculleus, Methanospirillum,* and *Methanosarcina*, though the latter can also perform acetoclastic methanogenesis. In the first-stage reactors, we observed almost exclusively a sole hydrogenotrophic methanogen, *Methanothermobacter* (Fig. 5). Methane production was very low in the first-stage reactors, but this suggests that the small amounts detected were due to hydrogenotrophic methanogenesis by this apparently low pH-tolerant methanogen. Additionally, the 3-day HRT would have caused the washout of the slow-growing acetoclastic methanogens, while hydrogenotrophic methanogens could retain their activity due to their higher growth rates [65].

## Conclusions

In summary, no strong differences were noted in biogas production or the TCOD converted to biogas for either the comparison of 55 vs. 65 °C first-stage reactors nor for the comparison of lowered HRT in the second-stage reactors. However, this suggests the potential for economic savings by running staged reactors with similar food waste feedstocks at a lower temperature (55 °C) to consume less energy and with a lower HRT (10 days) which requires less reactor tank volume. Microbial diversity was higher in the mesophilic stage, and this included a higher richness of hydrogenotrophic and acetoclastic methanogens. The distinct microbial communities in each reactor could be explained by the unique set of environmental conditions that resulted from operational control of temperature and HRT. In fact, just two parameters, pH and ammonium concentration, recapitulated the separation seen in the microbial community composition. We also identified taxa whose abundance changed as a result of the experimental conditions established in each reactor, like for instance *Ruminococcus*, which comprised 50% of the community in the 55 °C TDS1 and TDS3, whereas *Thermoanaerobacterium* and *Coprothermobacter* dominated in the 65 °C TDS2 and TDS4.

## Additional files

**Additional file 1: Image S1.** Photograph of representative reactor vessels in the two-stage digestion system used in this study. The system consists of a smaller-volume, thermophilic, acidogenic reactor (right), and a larger-volume, mesophilic, methanogenic reactor (left) as described in the methods.

**Additional file 2: Figure S1.** Schematic of the reactor systems used in this experiment. The operational parameters that varied between experimental period I and II are indicated in the schematic.

**Additional file 3: Spreadsheet S1.** Reactor process metadata corresponding to all microbiological sampling timepoints. Note that these values are point measurements and span a range of timepoints distinct from those in Table 2.

**Additional file 4: Protocol S1.** DNA extraction protocol used in this study to obtain template for 16S amplification and sequencing from the reactor samples.

**Additional file 5: Figure S3.** Oligotyping bipartite network showing nodes as oligotypes (purple) samples according to colors in the figure legend. The thickness of edges (lines) increases with increased relative abundance of the oligotype within a sample. Edge colors correspond to the sample colors in the legend. Spatial proximity of sample nodes indicates higher proportion of shared oligotypes.

**Additional file 6: Figure S4.** The relative abundance of bacterial phyla for each reactor timepoint. The colored bars correspond to different phyla as indicated in the legend on the right side, and only those phyla with > 1% relative abundance listed. Length of the bar indicates the proportion of the phylum within the overall community.

**Additional file 7: Figure S2.** Rarefaction curves for observed OTU_0.97_ (a, c) and Shannon diversity (b, d) as determined for reactor temperature (a, b) and reactor system (c, d).

**Additional file 8: Figure S5.** Principal Components Analysis (PCA) plot (A) based on the data for 6 of the process variables that are indicated by vectors in the plot and scree plot (B) of the percent of variation explained by each Principle Component. The legend at the top of panel A indicates the colors and symbols that correspond to each reactor. The percentages given on the axis labels in panel A indicate the variation explained by the axis and corresponds to values in the scree plot (B).

**Additional file 9: Figure S6.** Boxplots of process parameter values by reactor for TCOD (a), PCOD (b), SCOD (c), pH (d), NH_4_^+^ (e), alkalinity (f), total VFA (g), FOS/TAC (h), volatile solids (i), 3-day mean biogas (j), acetate (k), propionate (l), and butyrate (m).

**Additional file 10: Figure S7.** Boxplots of taxa percent relative abundances by reactor for Euryarchaeota (a), Firmicutes (b), Bacteroidetes (c), WWE1 (d), WPS-2 (e), WS6 (f), OP9 (g), *Methanothermobacter* (h), *Coprothermobacter* (i), *Ruminococcus* (j), and *Thermoanaerobacterium* (k).

## Abbreviations

HRT: hydraulic retention time
TCOD: total chemical oxygen demand
MDS: mesophilic digestion system
TDS: thermophilic digestion system
VFA: volatile fatty acids
OLR: organic loading rate
OTU: operational taxonomic unit
COD: chemical oxygen demand
SCOD: soluble chemical oxygen demand
VS: volatile solids
DS: digestion system
AD: anaerobic digestion
