# Supplementary figures and images for: The effect of temperature and retention time on methane production and microbial community composition in staged anaerobic digesters fed with food waste
Source: Biotechnol Biofuels. 2017 Dec 14;10:302. doi: 10.1186/s13068-017-0989-4 (PMC5729454; doi:10.1186/s13068-017-0989-4)

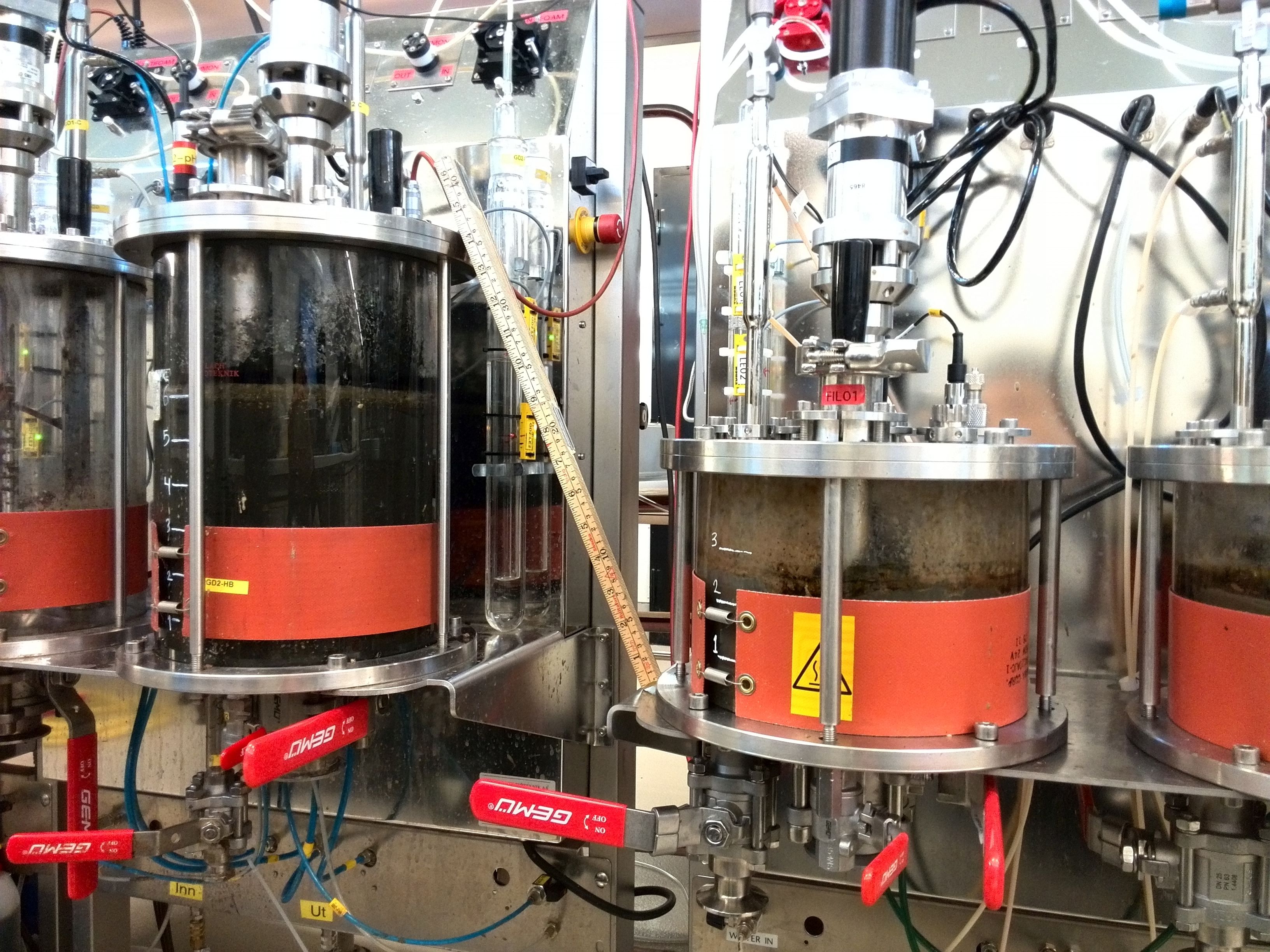

Supplement: Supplementary file 1 — Additional file 1: Image S1. Photograph of representative reactor vessels in the two-stage digestion system used in this study. The system consists of a smaller-volume, thermophilic, acidogenic reactor (right), and a larger-volume, mesophilic, methanogenic reactor (left) as described in the methods. [file 13068_2017_989_MOESM1_ESM.jpg]

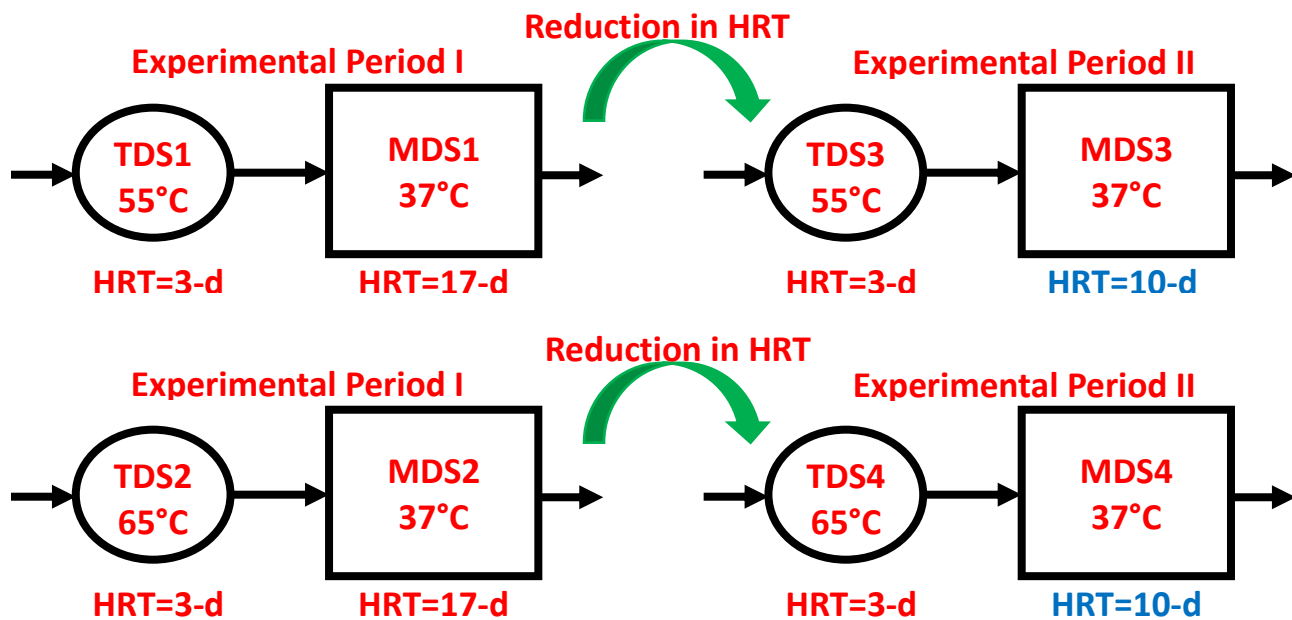

Supplement: Supplementary file 2 — Additional file 2: Figure S1. Schematic of the reactor systems used in this experiment. The operational parameters that varied between experimental period I and II are indicated in the schematic. [file 13068_2017_989_MOESM2_ESM.pdf]

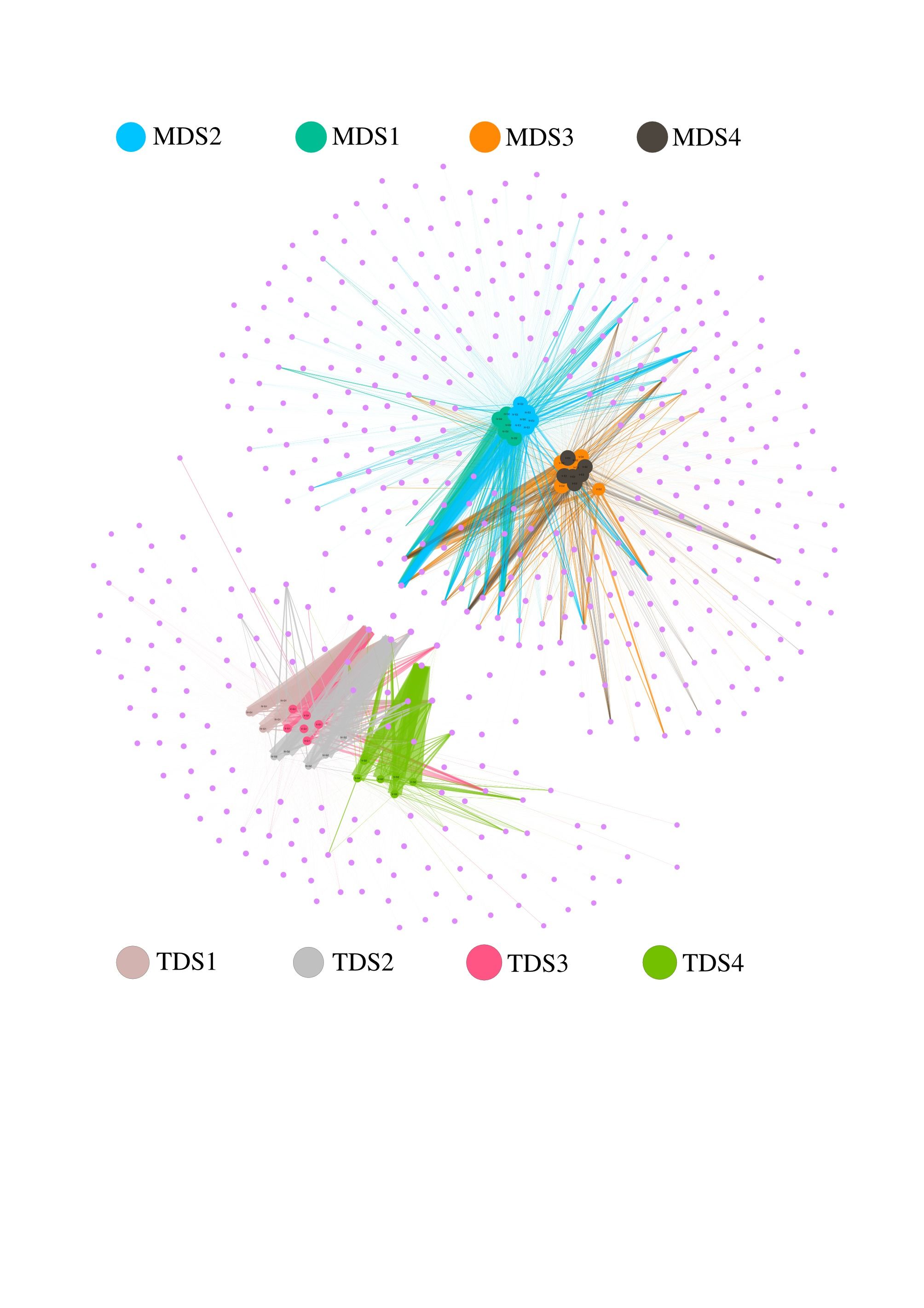

Supplement: Supplementary file 5 — Additional file 5: Figure S3. Oligotyping bipartite network showing nodes as oligotypes (purple) samples according to colors in the figure legend. The thickness of edges (lines) increases with increased relative abundance of the oligotype within a sample. Edge colors correspond to the sample colors in the legend. Spatial proximity of sample nodes indicates higher proportion of shared oligotypes. [file 13068_2017_989_MOESM5_ESM.jpg]

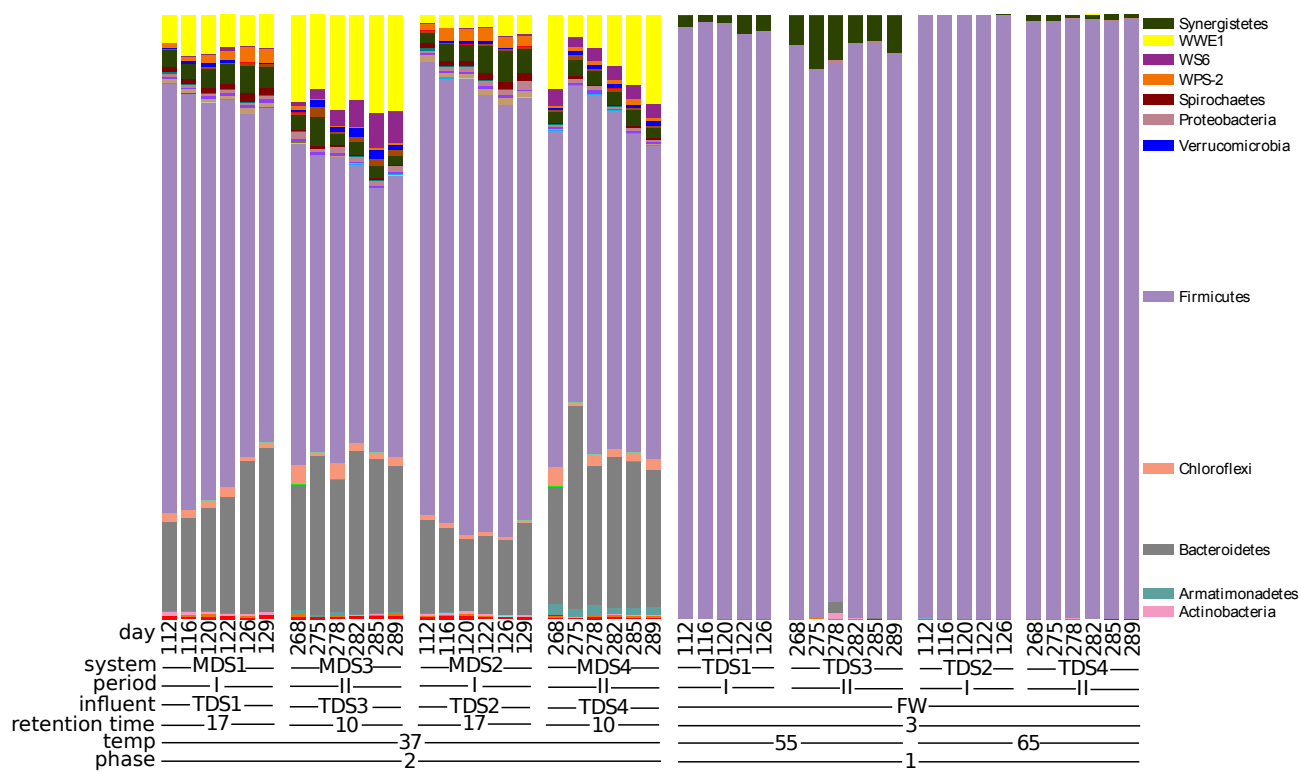

Supplement: Supplementary file 6 — Additional file 6: Figure S4. The relative abundance of bacterial phyla for each reactor timepoint. The colored bars correspond to different phyla as indicated in the legend on the right side, and only those phyla with > 1% relative abundance listed. Length of the bar indicates the proportion of the phylum within the overall community. [file 13068_2017_989_MOESM6_ESM.pdf]

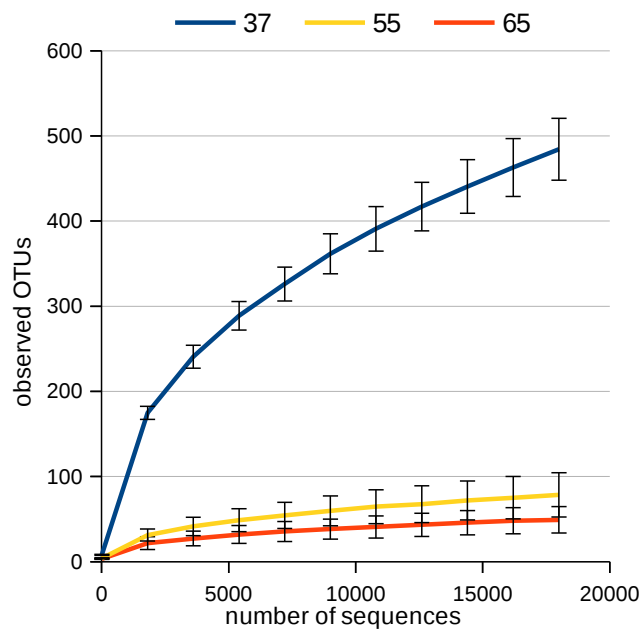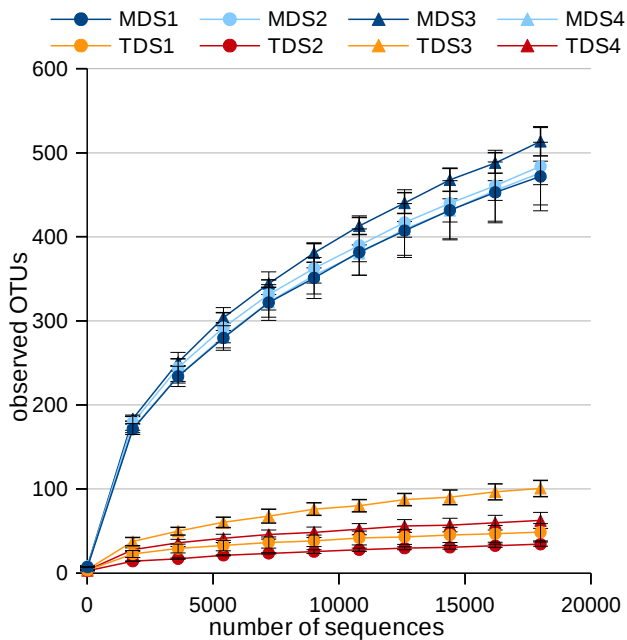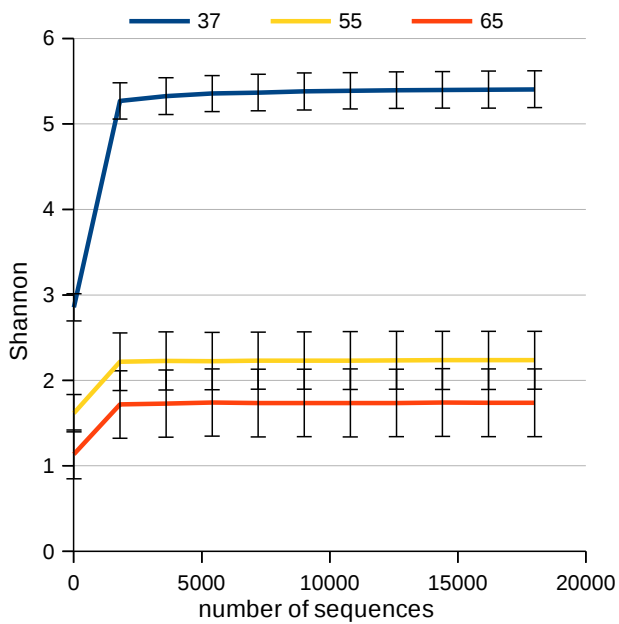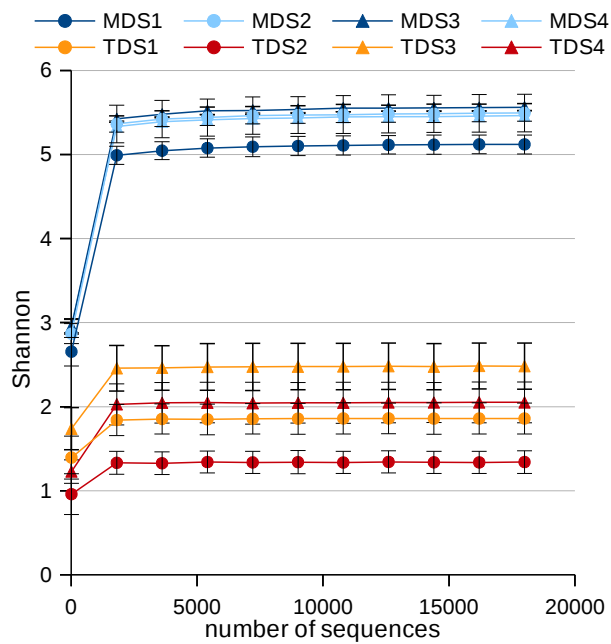

Supplement: Supplementary file 7 — Additional file 7: Figure S2. Rarefaction curves for observed OTU0.97 (a, c) and Shannon diversity (b, d) as determined for reactor temperature (a, b) and reactor system (c, d). [file 13068_2017_989_MOESM7_ESM.pdf]

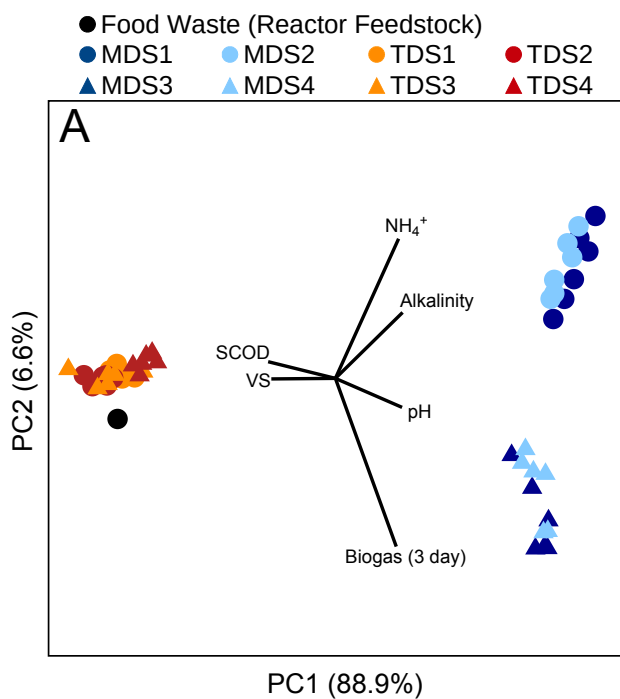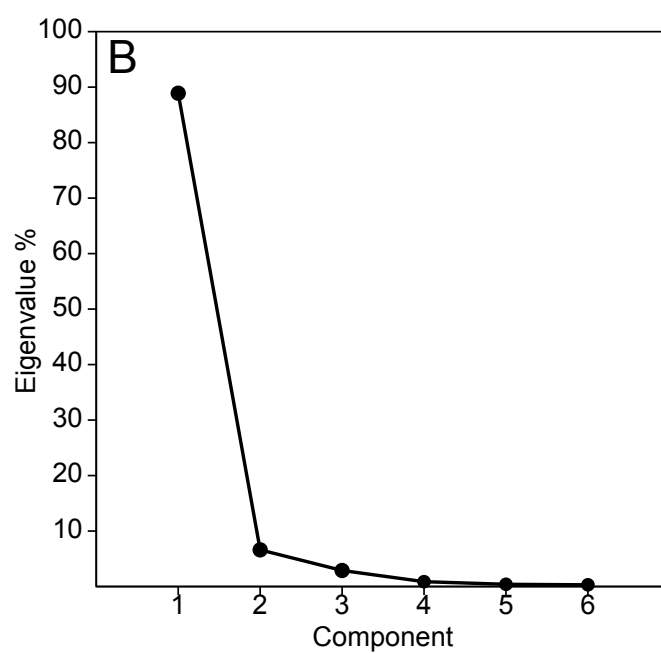

Supplement: Supplementary file 8 — Additional file 8: Figure S5. Principal Components Analysis (PCA) plot (A) based on the data for 6 of the process variables that are indicated by vectors in the plot and scree plot (B) of the percent of variation explained by each Principle Component. The legend at the top of panel A indicates the colors and symbols that correspond to each reactor. The percentages given on the axis labels in panel A indicate the variation explained by the axis and corresponds to values in the scree plot (B). [file 13068_2017_989_MOESM8_ESM.pdf]

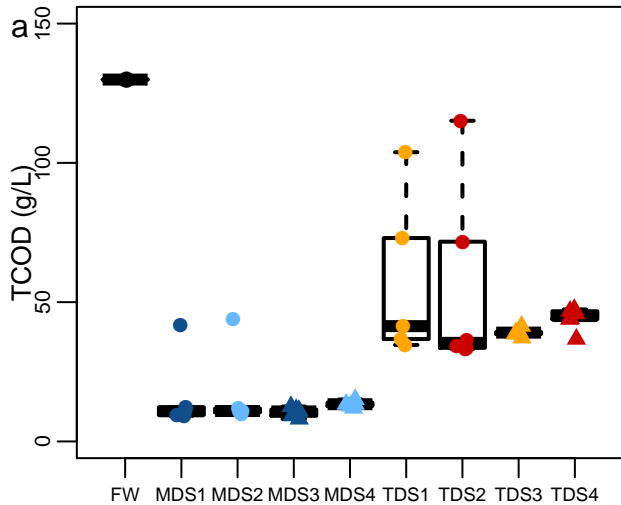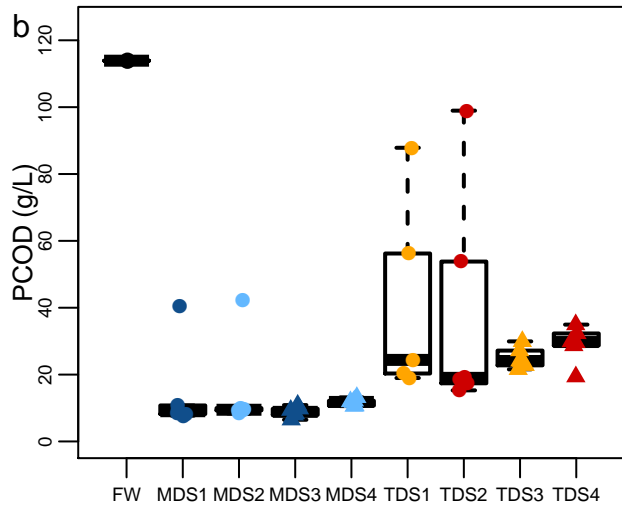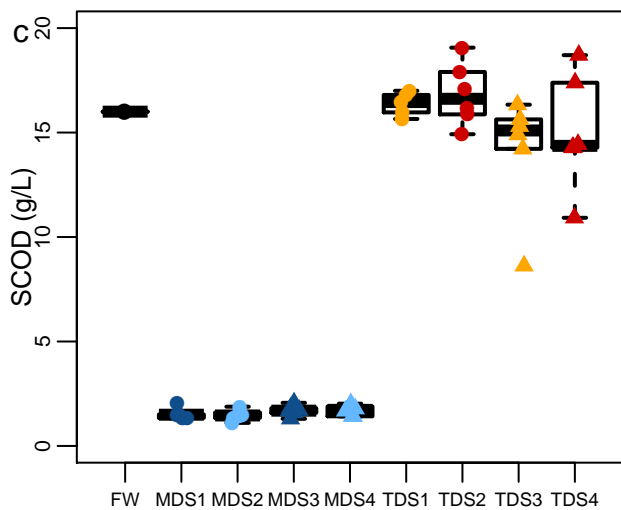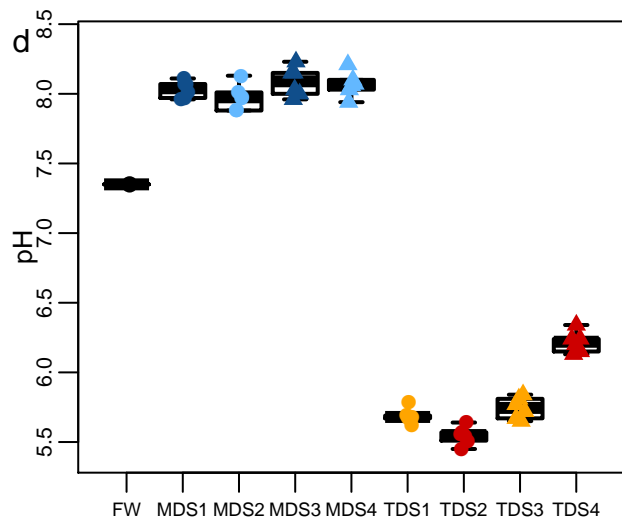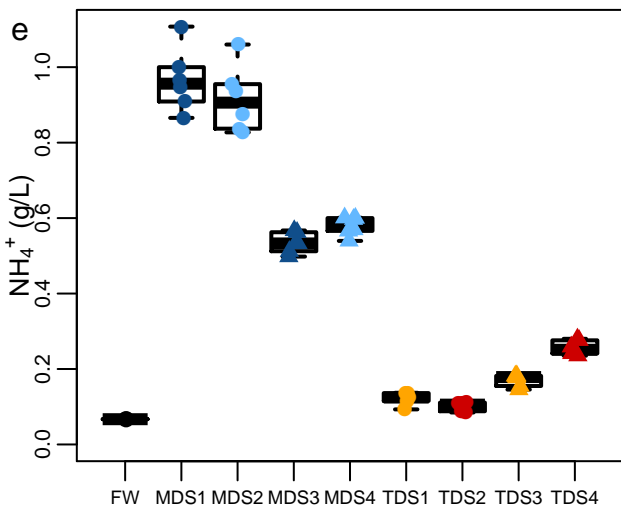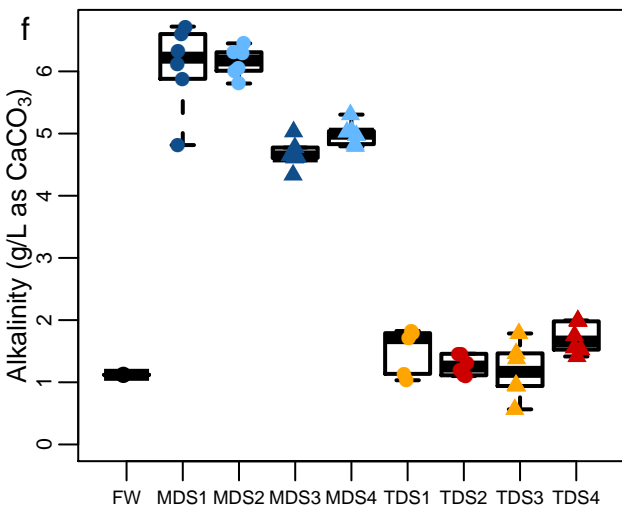

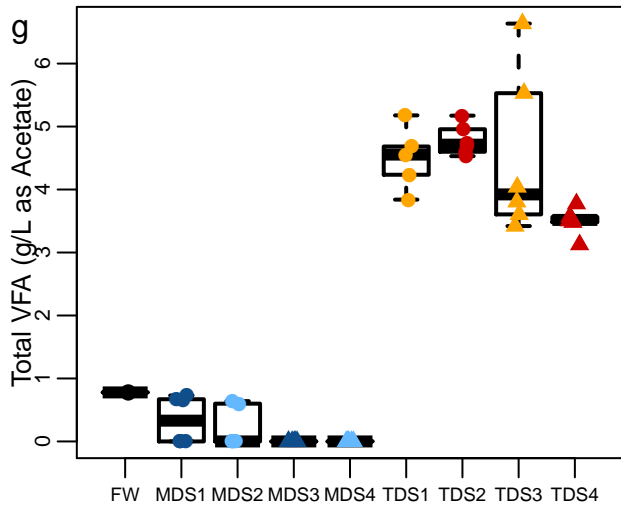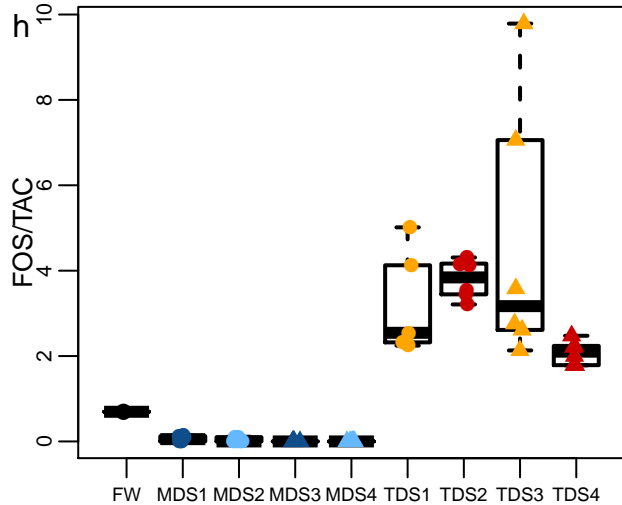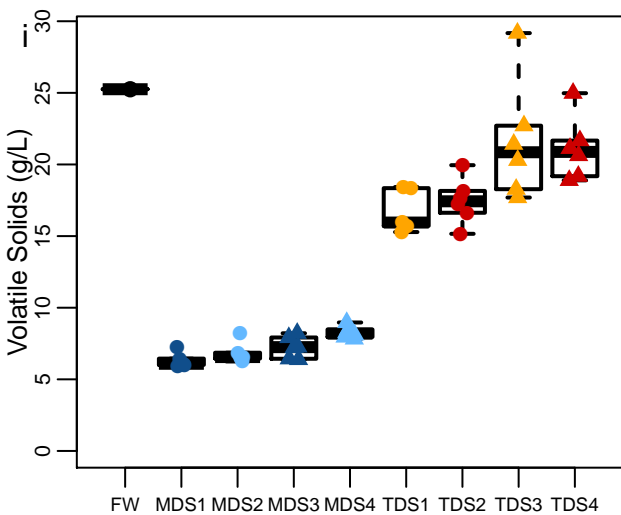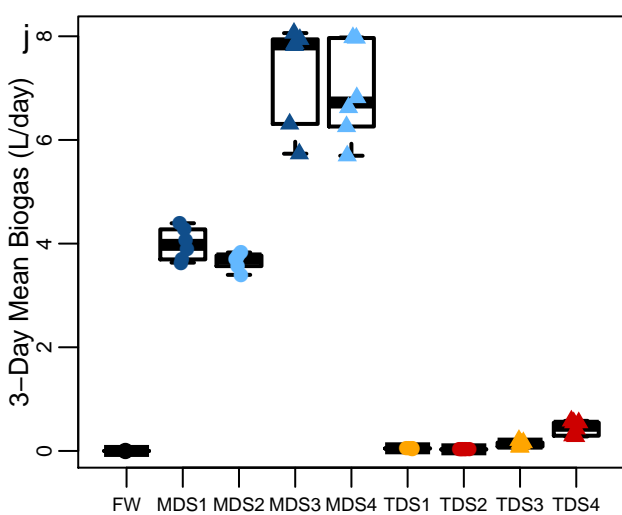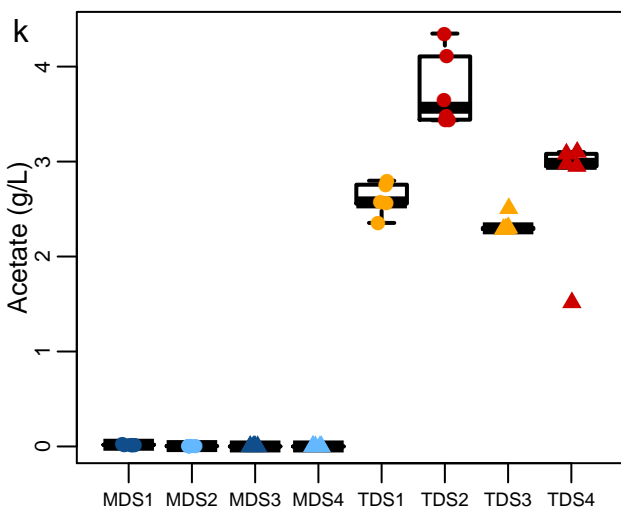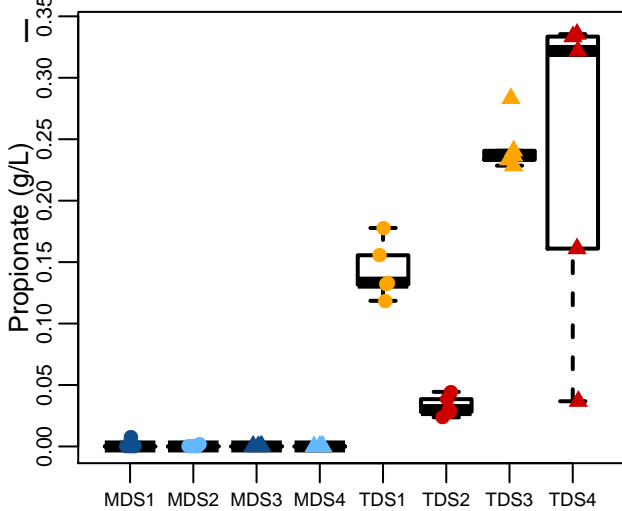

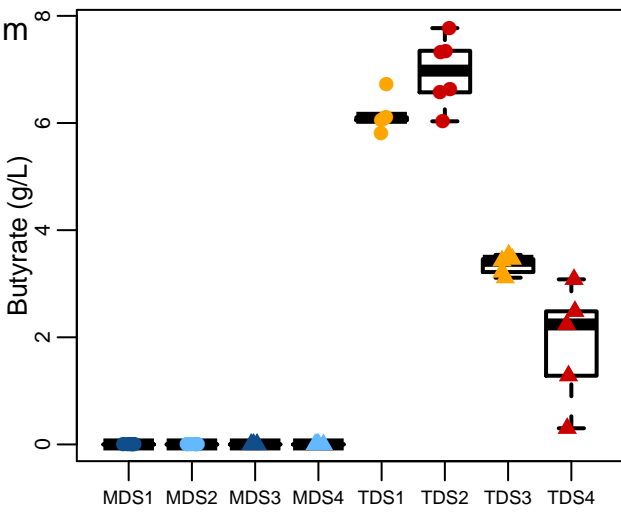

Supplement: Supplementary file 9 — Additional file 9: Figure S6. Boxplots of process parameter values by reactor for TCOD (a), PCOD (b), SCOD (c), pH (d), NH4 + (e), alkalinity (f), total VFA (g), FOS/TAC (h), volatile solids (i), 3-day mean biogas (j), acetate (k), propionate (l), and butyrate (m). [file 13068_2017_989_MOESM9_ESM.pdf]

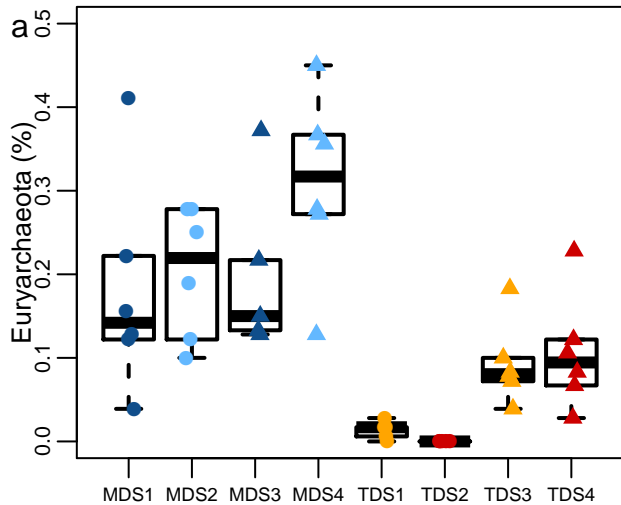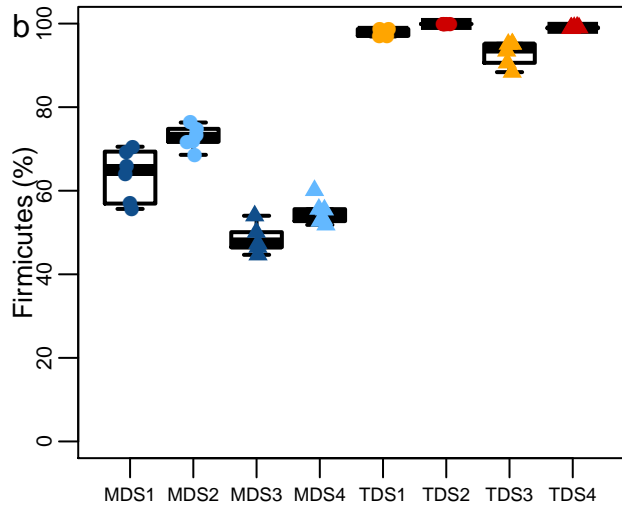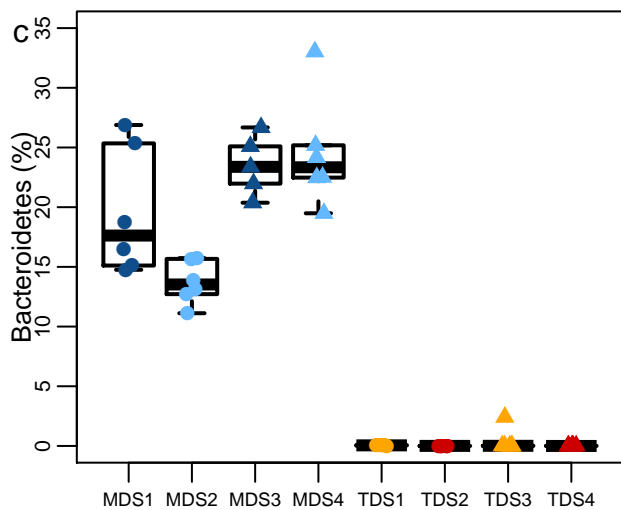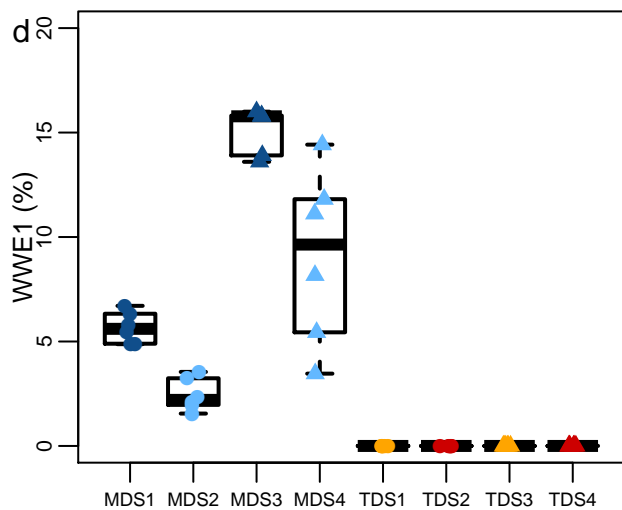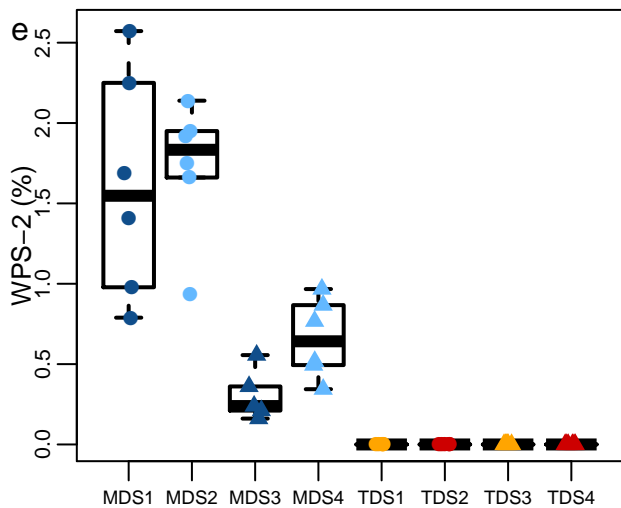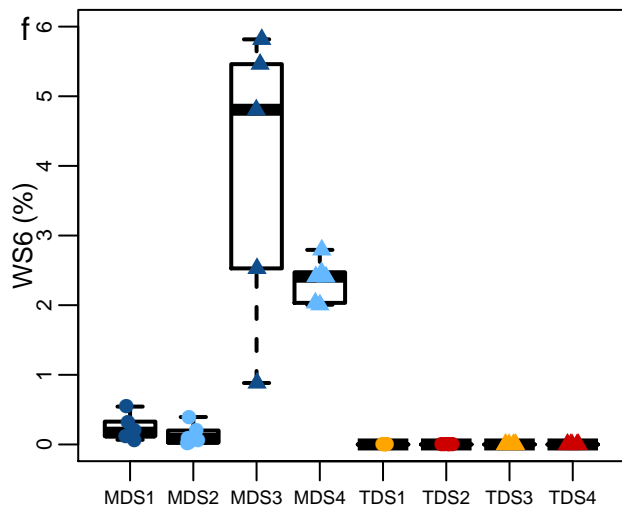

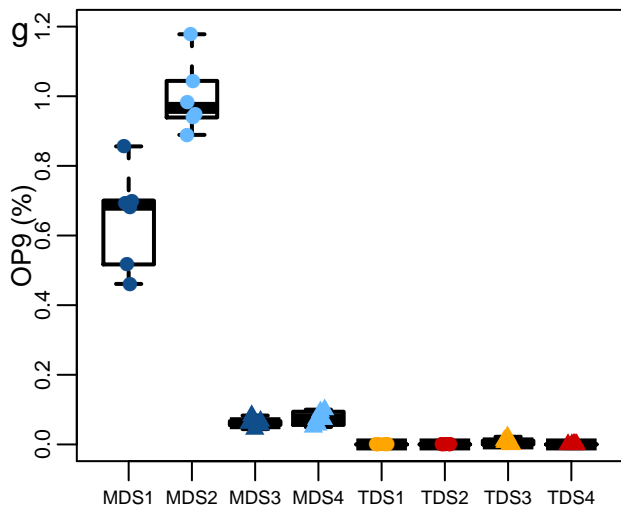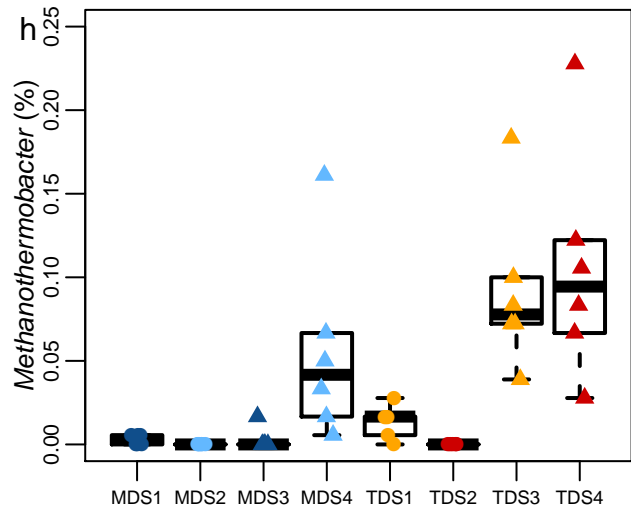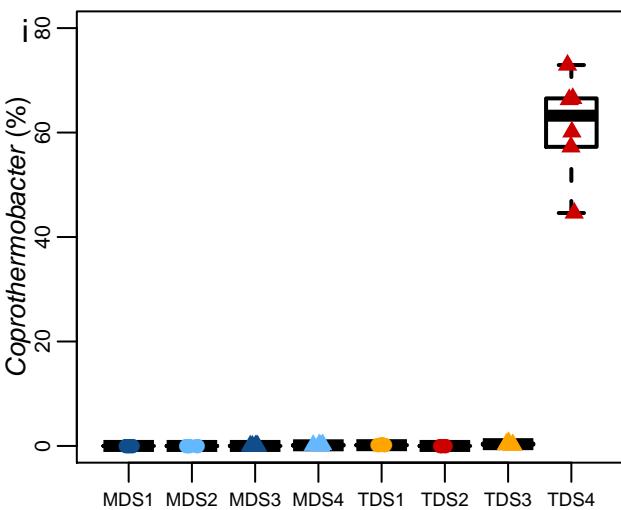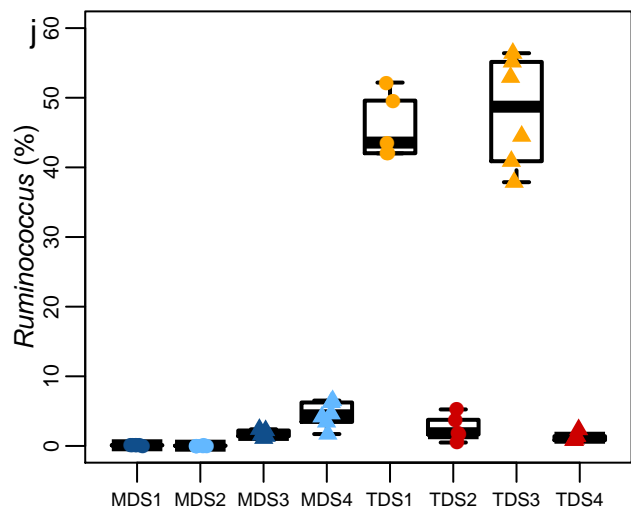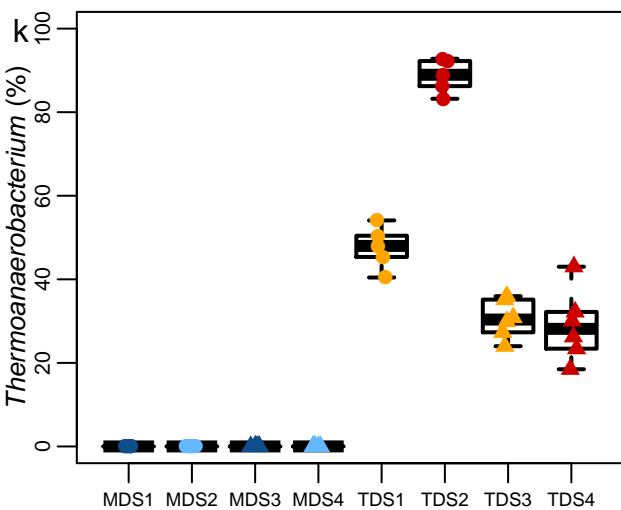

Supplement: Supplementary file 10 — Additional file 10: Figure S7. Boxplots of taxa percent relative abundances by reactor for Euryarchaeota (a), Firmicutes (b), Bacteroidetes (c), WWE1 (d), WPS-2 (e), WS6 (f), OP9 (g), Methanothermobacter (h), Coprothermobacter (i), Ruminococcus (j), and Thermoanaerobacterium (k). [file 13068_2017_989_MOESM10_ESM.pdf]
