# Supplementary material for: The effect of temperature and retention time on methane production and microbial community composition in staged anaerobic digesters fed with food waste
Source: Biotechnol Biofuels. 2017 Dec 14;10:302. doi: 10.1186/s13068-017-0989-4 (PMC5729454; doi:10.1186/s13068-017-0989-4)
Supplement: Supplementary file 4 — Additional file 4: Protocol S1. DNA extraction protocol used in this study to obtain template for 16S amplification and sequencing from the reactor samples. [file 13068_2017_989_MOESM4_ESM.docx]

**DNA extraction protocol for rumen and reactor samples**

Written by **John Christian Gaby** on **February 19, 2016**

Protocol introduced to the PEP group at NMBU by **Phil Pope**

The following protocol is derived from work published by Rosewarne et al.^1^ whereby they evaluated several approaches for extraction of DNA from herbivore gut samples. Their protocol derives from a previous publication by Yu and Morrison^2^ which in turn examined several approaches for extraction of DNA from digesta and fecal samples, and which is the origin of the repeated bead beating and column purification (RBB + C) buffer, though in the present protocol no bead beating or column purification is used but rather the RBB + C buffer is used to lyse pelleted cells and protect the DNA from degradation. This protocol has been used with success for both rumen digesta and biogas reactor samples for amplicon and shotgun metagenomics sequencing. This protocol yields high molecular weight DNA suitable for fosmid library construction.

**The following materials will be needed**:

2 mL microcentrifuge tubes

wide-orifice 1000 µL pipette tips

standard orifice 1000 µL pipette tips

standard orifice 10-100 µL pipette tips

appropriately-sized, nuclease-free, sterile reagent containers

filters to filter-sterilize the reagent solutions

**The following reagents will be needed**:

**Dissociation solution (pH 2.0)**

|  |  | **amount to add (ml)** | | | |
| --- | --- | --- | --- | --- | --- |
| **component** | **final concentration** | **100 extractions** | **200 extractions** | **500 extractions** | **1000 extractions** |
| methanol | 1% (v/v) | 2 | 4 | 10 | 20 |
| tert-butanol | 1% (v/v) | 2 | 4 | 10 | 20 |
| tween 80 | 0.1% (v/v) | 0.2 | 0.4 | 1.0 | 2.0 |
| nuclease-free water | --- | 200 | 400 | 1000 | 2000 |

2 ml of dissociation solution are needed per extraction. Filter sterilize this solution and store at 4 °C.

**Cell wash buffer**

|  |  | **amount to add (g)** | | | |
| --- | --- | --- | --- | --- | --- |
| **component** | **final concentration** | **100 extractions** | **200 extractions** | **500 extractions** | **1000 extractions** |
| tris-HCl | 10 mM | 0.1576 | 0.3152 | 0.788 | 1.576 |
| NaCl | 1 M | 5.8443 | 11.6886 | 29.2215 | 58.443 |
| nuclease-free water | --- | 100 | 200 | 500 | 1000 |

This solution may be autoclaved. Store at 4 °C.

**RBB+C buffer**

|  |  | **amount to add (g)** | | | |
| --- | --- | --- | --- | --- | --- |
| **component** | **final concentration** | **100 extractions** | **200 extractions** | **500 extractions** | **1000 extractions** |
| NaCl | 500 mM | 2.92 | 5.84 | 14.6 | 29.2 |
| tris-HCl | 50 mM | 0.788 | 1.576 | 3.94 | 7.88 |
| EDTA | 50 mM | 1.86 | 3.72 | 9.3 | 18.6 |
| SDS | 4% (w/v) | 4 | 8 | 20 | 40 |
| nuclease-free water | --- | 100 | 200 | 500 | 1000 |

Do not autoclave this solution since the SDS will foam. Heat the solution to 70 °C to dissolve the SDS before each use if precipitated. Filter sterilize and store at 4 °C.

**CTAB solution (CTAB is Cetyl Trimethyl Ammonium Bromide)**

|  |  | **amount to add (g)** | | | | | |
| --- | --- | --- | --- | --- | --- | --- | --- |
| **component** | **final concentration** | | **100 extractions** | | **200 extractions** | **500 extractions** | **1000 extractions** |
| NaCl | 0.7 M | 0.409 | | 0.818 | | 2.045 | 4.09 |
| CTAB | 10% (w/v) | 1.0 | | 2.0 | | 5.0 | 10.0 |
| nuclease-free water | --- | 10 | | 20 | | 50 | 100 |

Add the CTAB to 10 mL water in a 15 ml conical tube and vortex until dispersed. Heat to 70 °C with occasional vortexing so that the CTAB doesn’t form a gelatinous pellet at the bottom of the tube. Store at 4 °C.

**Cell Dissociation**

1. Homogenize the sample and use a wide-orifice, 1000 µL pipette to transfer approximately 0.5 to 1.0 g of each disgesta sample into 2.0 ml tubes.
2. Centrifuge at 10,000 rcf for 2 minutes and dispose of the supernatant.
3. Resuspend the pelleted digesta in 500 µl dissociation solution.
4. Vortex for 30 s.
5. Centrifuge at 100 to 500 rcf for 30 s at room temperature.
6. Transfer the cell-containing supernatant to new collection tube.
7. Repeat steps 3 to 6 for a total of 4 times to increase the amount of cell-containing supernatant.
8. Pellet the cells by centrifuging the collected supernatant volume (~2.0 ml) at 10,000 rcf for 5 minutes at room temperature.
9. Dispose of the supernatant.
10. Wash the cells in 1 ml cell wash buffer.
11. Centrifuge at 100 to 500 rcf for 30 s at room temperature to remove any remaining large particulates.
12. Transfer the cell-containing supernatant to fresh tube.
13. Centrifuge at 10,000 rcf for 5 to 20 minutes at room temperature and pipet off the supernatant. The remaining cell pellet should weigh ~200 mg.

**DNA Extraction**

1. Resuspend the cell pellet into 1 ml RBB+C buffer by vortexing for ~5 s.
2. Incubate for 20 minutes at 70 °C while **mixing the tube every 5 minutes** (4 to 6 inversions each time).
3. Add 40 µl of 5 M NaCl to bring the NaCl concentration to ~0.7 M.
4. Divide the suspension volume into 2 tubes by adding 520 µl to a new 1.5 ml tube.
5. Add to each tube 52 µl of CTAB solution.
6. Incubate for 10 minutes at 70 °C.
7. Add 1 volume chloroform, mix by inversion and centrifuge at 10,000 rcf for 10 minutes at room temperature.
8. Transfer the upper phase (~500 µl) to a new 1.5 ml tube and repeat steps 5-8 for 2 to 3 additional times until the upper phase becomes clear. For the final transfer, consolidate the 2 separate aqueous phase volumes from a sample into a single 2 ml tube.
9. Add 1 volume (~750 to 800 µl) of phenol:chloroform:isoamylalcohol (25:24:1).
10. Mix by inversion and spin at 10,000 rcf for 10 minutes.
11. Transfer the upper phase and precipitate the DNA by using 1 volume isopropanol at -20 °C. (Alternatively, 2 volumes ethanol may be used, but the combined sample + ethanol volume would typically exceed the 2 ml volume of the microcentrifuge tube).
12. Allow the DNA precipitate overnight at -20 °C in a freezer.
13. Centrifuge for 10 minutes at 14,000 rcf.
14. Pour off the alcohol while being careful not to lose the pellet.
15. Wash the pellet with -20 °C, 70% ethanol (~100 µL, more if it is a big pellet).
16. Centrifuge for 10 minutes at 14,000 rcf at room temperature, then carefully pour off the ethanol.
17. Let the pellet air dry several hours or overnight at room temperature.
18. Dissolve the DNA in 100 µl water or TE buffer.

**Additional Notes**

- The DNA may be spooled onto a loop, washed in 70% ethanol, dried, and resuspended in 100 µL TE.
- An aliquot may be checked on a 0.7% gel run at 70v for 1 hour against a 36kb marker (100 ng/µL) ran also to estimate concentration.
- Previous results indicate recovery of high molecular weight DNA ≥36 kb.
- DNA may be additionally quantified using QUBIT with 1 to 20 µL of sample.
- Previous results indicate DNA concentrations in the range 1.2-1.8 µg/µL.

**References**

^1^ Rosewarne CP, Pope PB, Denman SE, McSweeney CS, O'Cuiv P, Morrison M (2011). High-yield and phylogenetically robust methods of DNA recovery for analysis of microbial biofilms adherent to plant biomass in the herbivore gut. Microb Ecol. 61(2): 448-454.

^2^ Yu Z, Morrison M (2004). Improved extraction of PCR-quality community DNA from digesta and fecal samples. Biotechniques. 36: 808–812.
